# Regulation of Human Trophoblast GLUT3 Glucose Transporter by Mammalian Target of Rapamycin Signaling

**DOI:** 10.3390/ijms160613815

**Published:** 2015-06-16

**Authors:** Jie Xu, Chunmei Lu, Jiao Wang, Ruotong Zhang, Xin Qian, Hui Zhu

**Affiliations:** Laboratory of Reproductive Endocrinology, Department of Physiology, Harbin Medical University, Harbin 150081, China; E-Mails: xujie@ems.hrbmu.edu.cn (J.X.); angel200202003@163.com (C.L.); wangj_84321@163.com (J.W.); qilinyehappy@163.com (R.Z.); qianxiaoniude@163.com (X.Q.)

**Keywords:** placenta, glucose transporter isofrom-3, mammalian target of rapamycin

## Abstract

Glucose transporter isoform-3 (GLUT3), one of the primary placental facilitative glucose transporters responsible for basal glucose transport, has a crucial role in glucose transport and fetal growth during early pregnancy. A GLUT3 mutation in mice has been reported to cause loss of early pregnancy or late-gestational fetal growth restriction. However, the underlying mechanisms that regulate the placental GLUT3 transporter in humans are largely unknown. In the present study, we used the JEG-3 human choriocarcinoma cell line, which resembles a first trimester placental model, to study the role of the mammalian target of rapamycin complex 1 (mTORC1) in the regulation of placental GLUT3. We combined rapamycin treatment and small interfering (si) RNA-mediated silencing approaches with mRNA and protein expression/localization studies to investigate the alteration of GLUT3 expression and localization following mTORC1 inhibition in JEG-3 trophoblasts. Inhibition of mTORC1 signaling by silencing raptor decreased GLUT3 mRNA expression (−41%) and protein expression (−50%). Similar effects were obtained in cells in which mTORC1 was inhibited by rapamycin. Immunofluorescence analysis revealed that GLUT3 expression was markedly reduced in the cell surface and cytoplasm of JEG-3 cells in response to mTORC1 silencing. Because placental mTORC1 activity and GLUT3 expression are decreased in human intrauterine growth restriction, our data suggested one possible mechanism for the abnormal fetal growth in this pregnancy complication.

## 1. Introduction

Inadequate nutrient supply from the mother to the fetus leads to abnormal intrauterine fetal growth, which may increase the risk for health problems, including obesity, diabetes and cardiovascular disease in both childhood and later life [1]. Glucose is the principal energy substrate for both the placenta and fetus and is essential for normal fetal metabolism and growth. The fetus has a very low capacity for glucose production, so glucose transport from the maternal circulation to the fetus by placental facilitated diffusion is the dominant mechanism by which the fetus acquires glucose [2]. Glucose uptake and transport by trophoblast cells are mediated by facilitative transporter proteins, primarily by glucose transporter isoform-1 (GLUT1) and isoform-3 (GLUT3). GLUT1 is a ubiquitous transporter found in all cells of the placenta; however, GLUT3 has a more restricted expression pattern, which suggests specialized functions for this protein. The GLUT3 isoform is expressed in cytotrophoblasts during early gestation but is localized primarily to the fetal vascular endothelium during late gestation [3,4]. This type of distribution pattern has important implications for glucose flux across the placenta. A glucose molecule must sequentially use both GLUT1 and GLUT3 to cross from maternal to fetal circulation [5], and the glucose supply to the fetus depends on the concentration gradient between mother and fetus [6,7]. Therefore, endothelial GLUT3 expression has been strongly implicated in enhancing the glucose flux across the placenta by decreasing the umbilical arterial glucose concentration, thereby increasing the glucose gradient between maternal circulation and fetal circulation and providing the driving force for transplacental transport [8]. However, more solid experimental evidence describing the role of placental GLUT3 is still necessary.

Placental GLUT expression is altered in pathological pregnancies such as those affected by intrauterine growth restriction (IUGR) or maternal diabetes, implicating changes in placental glucose transporters in altered fetal growth. For example, GLUT1 expression is increased in the basal membrane in diabetic pregnancies that correlates with a large infant [9,10]. In contrast, a decrease in placental GLUT1 expression has been shown in IUGR concomitant with reduced fetal growth [11]. The levels of placental GLUT3 expression appear to be unaffected by maternal diabetes or IUGR in humans. However, a reduction of the placental GLUT3 levels has been demonstrated in experimentally induced IUGR in mice [12]. Furthermore, birth weight corresponded to placental weight, indicates that the total amount of GLUT3 is increased in macrosomia and reduced in IUGR [13]. A recent study in humans showed that placental GLUT3 expression decreases across gestation with a markedly reduced expression towards term [3], and GLUT3 mutations in mice have been demonstrated to cause early losses of pregnancy or late-gestational fetal growth restriction [14,15]. Collectively, these findings indicate a greater role for this protein in glucose transport and fetal growth during early pregnancy. Although these changes in GLUT3 expression are important for fetal growth, the regulatory mechanisms controlling these alterations are still poorly understood.

The mammalian target of rapamycin (mTOR) signaling pathway has recently been suggested to be a nutrient sensor in the placenta, controlling fetal growth by regulating placental growth and nutrient transport [16]. mTOR is a ubiquitously expressed serine/threonine protein kinase that exists in two complexes, mTOR complexes 1 (mTORC1) and 2 (mTORC2) [17,18]. One key difference between the two complexes is that mTOR associates with the protein raptor (regulatory associated protein of mTOR) in mTORC1 and with rictor (rapamycin-insensitive companion of mTOR) in mTORC2. Placental mTORC1 activity is decreased in human IUGR [19,20], and it is speculated to be activated in diabetes associated with increased placental nutrient availability. Currently, mTORC1 is well established as a modulator of amino acid (AA) transfer across the placenta. However, very little is known regarding the regulatory role of mTORC1 signaling in placental glucose transport, especially with respect to placental GLUT3 expression and localization.

Considering that placental mTORC1 activity and GLUT3 expression are decreased in intrauterine growth restriction and that placental GLUT3 mutation has been shown to cause late-gestational fetal growth restriction, we tested one of the possible mechanisms for the development of abnormal fetal growth in this pregnancy complication: the decrease of placental GLUT3 levels resulted from inhibition of mTORC1 signaling may be one important mechanism in fetal growth restriction. To this end, the mTORC1 signaling pathway was inhibited in JEG-3 choriocarcinoma cells by rapamycin, a specific mTORC1 inhibitor, or by gene silencing targeting raptor, a key component of mTORC1, and subsequently, the mRNA expression, protein expression and localization of GLUT3 were measured.

## 2. Results and Discussion

### 2.1. Validation of mTORC1 Pathway Inhibition

**Figure 1:**
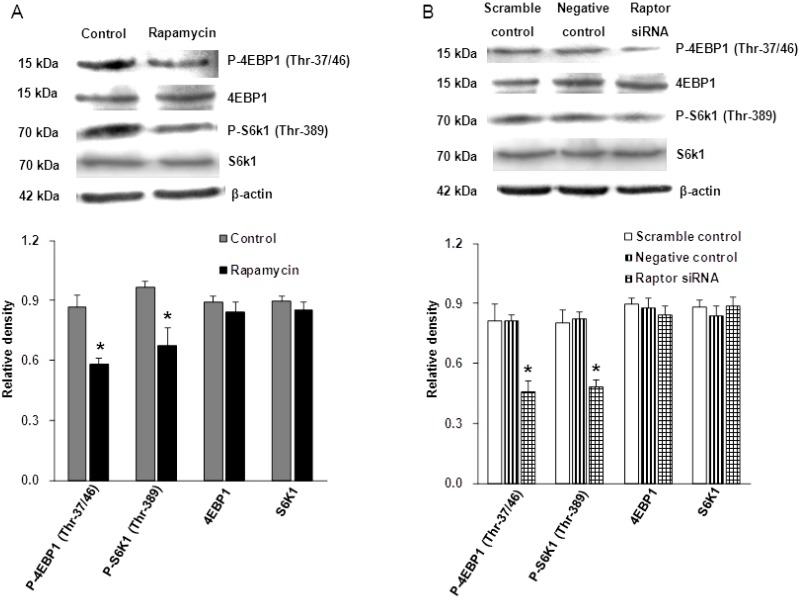
Effect of rapamycin and raptor silencing on mTORC1 activity. (**A**) Representative western blot analysis of phosphorylated S6k1 (Thr-389) and phosphorylated 4EBP1 (Thr-37/46) expression in the cell lysates of 100 nM rapamycin treated and control cells. Rapamycin significantly reduced the expression of phosphorylated S6k1 (Thr-389) and phosphorylated 4EBP1 (Thr-37/46), whereas it had no effect on total S6K1 and total 4EBP1 expression. Values are present as mean ± SD. *n* = 4, * *p* < 0.05 *versus* (*vs.*) control by Mann–Whitney *U*-test; (**B**) Representative western blot analysis of phosphorylated S6k1 (Thr-389) and phosphorylated 4EBP1 (Thr-37/46) expression in the cell lysates of the scramble control, negative control and raptor silenced cells. Phosphorylation of S6k1 (Thr-389) and 4EBP1 (Thr-37/46) was significantly decreased in the raptor silenced cells, but total S6K1 and total 4EBP1 expression were not changed. Values are present as mean ± SD. *n* = 4, * *p* < 0.05 *vs.* scramble control and negative control by Kruskal-Wallis test followed by Mann-Whitney *U*-test.

**Figure 2:**
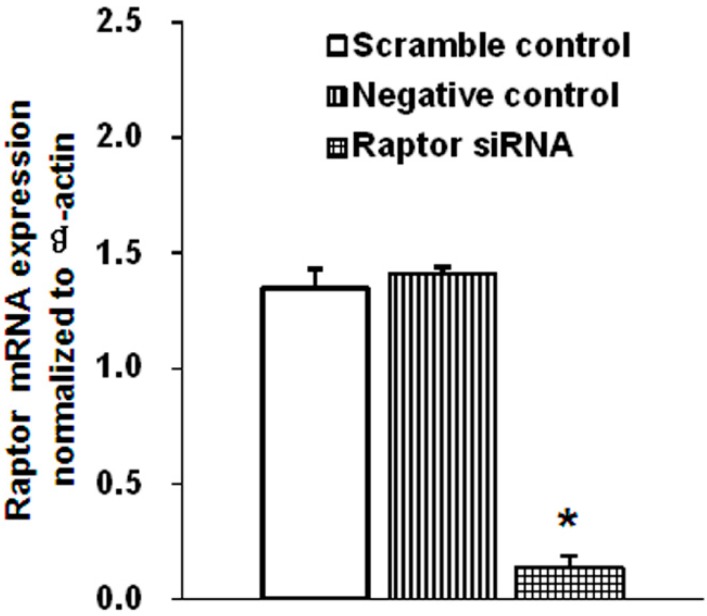
Raptor mRNA expression was markedly decreased after raptor silencing. Quantitative-PCR analysis showed that raptor gene was significantly silenced in the cells transfected with raptor siRNA. Values are present as mean ± SD. *n* = 6, * *p* < 0.01 *vs.* scramble control and negative control by Kruskal-Wallis test followed by Mann-Whitney *U*-test.

To confirm that both rapamycin and raptor siRNA reduced the mTORC1 activity in JEG-3 cells, the phosphorylation states of the ribosomal protein S6 kinase 1 (S6K1) and the eukaryotic translation factor 4E-bingding protein (4E-BP1), two functional readouts for mTOR activity, were measured. As shown in Figure 1A, the phosphorylation of S6K1 at Thr-389 was reduced by 30% (*n* = 4, *p* = 0.021) and phosphorylation of 4E-BP1 at Thr-37/46 was reduced by 33% (*n* = 4, *p* = 0.021) in the cells treated with rapamycin (Figure 1A). No changes were observed in the total 4E-BP1 (*n* = 4, *p* = 0.149) and S6K1expression (*n* = 4, *p* = 0.304) in rapamycin-treated cells compared with the control cells (Figure 1A). Similarly, following transfection to silence raptor, the expression levels of phosphorylated S6K1 (Thr-389) and phosphorylated 4E-BP1 (Thr-37/46) were significantly decreased by 42% and 44% (*n* = 4, *p* = 0.02), respectively (Figure 1B). The total 4E-BP1 (*n* = 4, *p* = 0.211) and S6K1 expression (*n* = 4, *p* = 0.225) were also unchanged in response to raptor silencing (Figure 1B). In addition, raptor gene expression (*n* = 6, *p* = 0.002) was markedly decreased after raptor siRNA transfection (Figure 2). These results demonstrate that both rapamycin treatment and the siRNA approach can reduce the activity of the trophoblast mTORC1 signaling pathway.

### 2.2. Effect of mTORC1 Inhibition on JEG-3 Cell Characteristics

**Figure 3:**
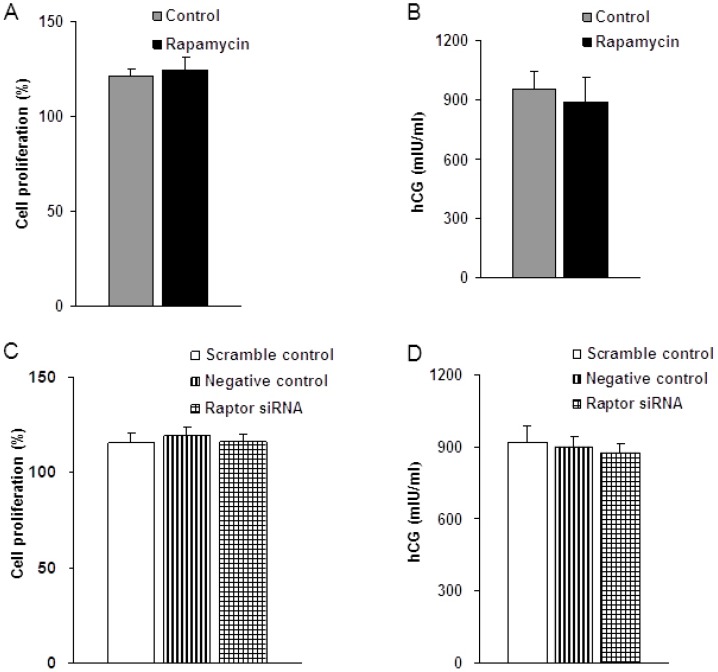
Rapamycin and raptor silencing does not affect JEG-3 cell characteristics. (**A**) Effect of rapamycin on JEG-3 cell proliferation; (**B**) Effect of rapamycin on the hCG secretion of JEG-3 cells. Values are mean ± SD. *n* = 10, *p* > 0.05 by Mann-Whitney *U*-test; (**C**) Effect of raptor silencing on JEG-3 cell proliferation; and (**D**) Effect of raptor silencing on the hCG production of JEG-3 cells. Values are means ± SD. *n* = 10, *p* > 0.05 by Kruskal-Wallis test.

To investigate the effects of rapamycin and raptor silencing transfection on the endocrine activity and cell growth of JEG-3 cells, human chorionic gonadotropin (hCG) measurement and cell proliferation assay were performed after 48 h in the cells incubated with rapamycin and raptor siRNA, scrambled siRNA (negative control), or transfection agent alone (scramble control). The results showed no significant differences in the cell proliferation (*n* = 10, *p* = 0.15) and hCG production (*n* = 10, *p* = 0.113) between cells incubated with media containing rapamycin and cells grown in control media (Figure 3A,B). Similarly, no significant difference in cell proliferation (*n* = 10, *p* = 0.197) and hCG secretion (*n* = 10, *p* = 0.739) was found in cells in which raptor had been silenced as compared to the negative control and scramble control (Figure 3C,D). These data indicate that inhibition of mTORC1 signaling did not adversely affect trophoblast cell endocrine activity and cell growth.

### 2.3. Effects of Rapamycin Treatment on GLUT3 Expression

**Figure 4:**
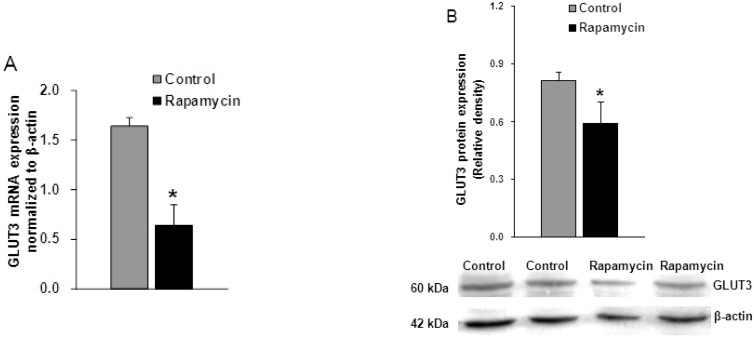
Effect of rapamycin on the expression of trophoblast GLUT3. (**A**) Effect of rapamycin on the mRNA expression of GLUT3. The expression of GLUT3 mRNA was down-regulated by 61% in the cells incubated with rapamycin, Values are mean ± SD. *n* = 5, * *p* < 0.01 *vs.* control by Mann-Whitney *U*-test; and (**B**) Effect of rapamycin on the protein expression of GLUT3. The GLUT3 protein level was reduced by 28% in rapamycin-treated cells, Values are mean ± SD. *n* = 4, * *p* < 0.05 *vs.* control by Mann-Whitney *U*-test.

To investigate the effect of mTORC1 inhibition on GLUT3 expression, we first used rapamycin, a specific mTORC1 inhibitor, to inhibit the trophoblast mTORC1 activity. Quantitative PCR and western blotting analyses were then conducted. As shown in Figure 3, the mRNA expression of GLUT3 was down-regulated (−60%) (*n* = 5, *p* = 0.009) in cells incubated with rapamycin (Figure 4A) compared with vehicle-treated cells. The levels of GLUT3 protein were also reduced by 28% (*n* = 4, *p* = 0.021) in rapamycin-treated cells compared with control cells (Figure 4B), which indicates that mTORC1 inhibition mediated by rapamycin treatment significantly reduced the trophoblast GLUT3 expression.

### 2.4. Effects of siRNA Targeting Raptor on GLUT3 Expression

**Figure 5:**
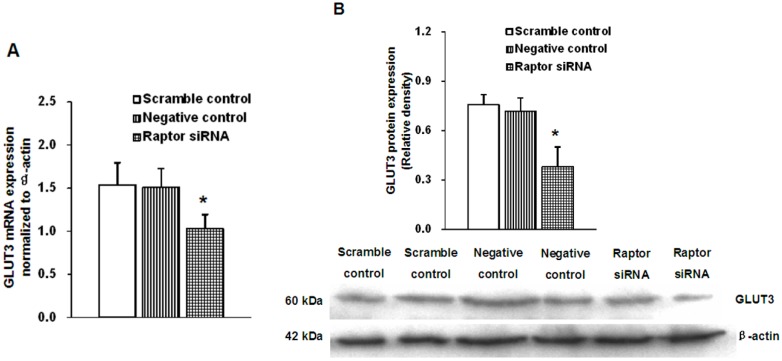
Effect of raptor silencing on the expression of trophoblast GLUT3. (**A**) Effect of raptor silencing on the expression of GLUT3 mRNA. The mRNA expression of GLUT3 was decreased by 41% in raptor siRNA cells, Values are mean ± SD. *n* = 5, * *p* < 0.05 *vs.* negative control and scramble control by Mann-Whitney *U*-test; (**B**) Effect of raptor silencing on the GLUT3 protein expression. The expression of GLUT3 protein was decreased by 50% in raptor-silenced cells, Values are mean ± SD. *n* = 4, * *p* < 0.05 *vs.* negative control and scramble control by Mann-Whitney *U*-test.

**Figure 6:**
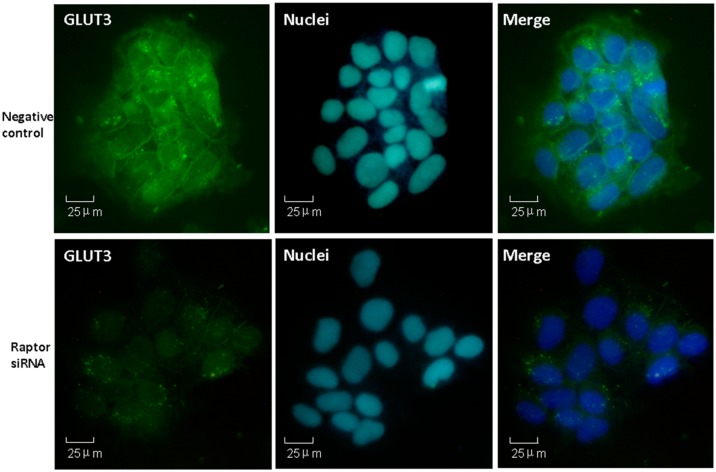
Cellular localization of GLUT3 protein expression in mTORC1 inhibited JEG-3 cells. JEG-3 cells were seeded in 24-well plates and transfected with scrambled (**upper**) or raptor-specific (**lower**) siRNA. GLUT3 protein expression (green) was visualized using immunofluorescence. Nuclei were counterstained with dye 4,6-diamido-2-phenylindole (DAPI) (blue).

We then examined the effect of mTORC1 inhibition mediated by raptor silencing on GLUT3 expression. Following knock-down of raptor, the GLUT3 mRNA and protein levels were significantly reduced by 41% (*n* = 5, *p* = 0.013) (Figure 4A) and 50% (*n* = 4, *p* = 0.023) (Figure 5B), respectively. To investigate the alteration of GLUT3 localization within cells in response to mTORC1 inhibition, we then used immunofluorescence to detect the cellular localization of GLUT3 in fixed cells. We observed that GLUT3 protein was present in the cell surface and the cytoplasm (Figure 6A), and in response to raptor silencing, the total expression of GLUT3 protein was reduced (Figure 6B) as compared to negative control cells (Figure 6A). Unexpectedly, GLUT3 was not relocated between the cell periphery and the interior in response to raptor silencing. These data suggested that silencing of trophoblast mTORC1 signaling decreased trophoblast GLUT3 protein expression without affecting its localization within the cells.

### 2.5. Discussion

The present study is the first to report that mTORC1 regulates the trophoblast glucose transporter isoform-3. We report that the mTORC1 signaling pathway is a powerful regulator of trophoblast GLUT3, as indicated by changes in the expression of GLUT3 mRNA and protein. Because altered placental GLUT3 expression will lead to abnormal fetal growth, our data suggest that dysregulation of placental mTORC1 activity plays a central role in the pathophysiology of abnormal fetal growth. Regulation of GLUT3 by trophoblast mTORC1 signaling may constitute another critical link between maternal nutrition availability and fetal survival and growth.

Trophoblast glucose transporter isoform-3 is one of the primary facilitative transport proteins that are responsible for basal glucose transport in the placenta. GLUT3 has been reported to have a critical life-sustaining role in embryonic development, because a homozygous null mutation of GLUT3 in mice may cause early pregnancy loss despite the presence of intact GLUT1 and a compensatory increase in AA transport [14,15]. In contrast, when another related isoform, GLUT8, was mutated, the homozygous null mutant mice are born normally [21]. More importantly, in the presence of a heterozygous null mutation of GLUT3, a decrease in GLUT3-mediated transplacental glucose transport to the fetus is observed, causing late-gestation fetal growth restriction [15]. However, the role and regulation of trophoblast GLUT3 in human early pregnancy are largely unelucidated. Therefore the human JEG-3 placental choriocarcinoma cell line, regarded as a first trimester placental model [22], was used to investigate regulation of GLUT3 expression. Because the JEG-3 cells are unable to morphologically differentiate and hence resemble undifferentiated cytotrophoblast cells, which makes it an appropriate *in vitro* model of the early gestational placenta [22,23].

mTOR, a ubiquitous protein, has been demonstrated to be present in JEG-3 cells. In addition, mTORC1 signaling has been reported to be decreased in human IUGR. In the present study, we combined rapamycin treatment and gene silencing targeting raptor to inhibit mTORC1 signaling in JEG-3 cells. Rapamycin is a specific mTORC1 inhibitor and raptor is a key component of mTORC1. Using these approaches, we verified that both rapamycin and gene silencing efficiently inhibited mTORC1 activity in JEG-3 cells, as indicated by the decreased phosphorylation of 4E-BP1 and S6K1. Furthermore, rapamycin and raptor silencing did not alter the characteristics of JEG-3 cells including their endocrine activity and cell growth activity, indicated by the lack of differences in hCG production and cell proliferation between the experimental and control groups.

There is extensive evidence supporting an important role for mTORC1 signaling in placental nutrient-sensing. For example, in cultured primary human trophoblast cells, mTORC1 is regulated by glucose, AA concentrations and growth factors [24]. Furthermore, these well-established stimuli of mTORC1 are usually altered in the placenta in common pregnancy complications. Specifically, in human IUGR, placental AA transporters including system A, system L and taurine transporters are down-regulated [25,26,27], and glucose and growth factors in the fetal and maternal circulation are reduced [28,29,30]. Thus, changes in the levels of local nutrients and growth factors would alter placental mTORC1 activity. In addition, trophoblast mTORC1 is well reported to modulate AA transfer across the placenta by regulating the activity of key AA transporters including System A and System L. In contrast to the well-known cellular effects of mTORC1 signaling, trophoblast mTORC1 regulation of AA transporters occurs mainly at the post-translational level by modulation of the translocation of specific transporter isoforms between the plasma membrane and the cell interior [31]. Although, mTORC1 activity has been shown to be decreased in glucose-deprived primary trophoblast cells [24], and the glucose concentrations of the fetus and the mother are reduced in IUGR, very little is known regarding the role of mTORC1 signaling pathway on the glucose transport across placenta. We demonstrated for the first time that trophoblast mTORC1 signaling modulates glucose transport isoform-3 at translational and transcriptional levels, markedly changing the expression of GLUT3 mRNA and protein in the JEG-3 cell line. We have hypothesized that mTORC1 inhibition may alter the sub-cellular localization of GLUT3 protein in JEG-3 cells. However, unexpectedly, immunofluorescence showed that unlike the specific AA transporter, the cellular localization of GLUT3 protein was not affected by mTORC1 inhibition in JEG-3 cells. We speculate that this discrepancy may be due to the use of different trophoblast models.

Because GLUT3 plays a greater role in early gestation, our report regarding its regulation by mTORC1 signaling in JEG-3 trophoblasts, an *in vitro* model of the early gestational placenta, provides a possible mechanism for the fetal growth restriction resulting from down-regulation of GLUT3. During late gestation, placental GLUT3 expression markedly decreases and localizes primarily to the fetal vascular endothelium [3,4]. Thus, the roles of GLUT3 and its regulation by mTORC1 signaling in late gestation might be quite different, which requires further studies.

## 3. Experimental Section

### 3.1. Cell Culture

The JEG-3 choriocarcinoma cell line was obtained from the Cell Bank of the Chinese Academy of Sciences. The cells were routinely maintained in Dulbecco’s Modified Eagle Media: Nutrient Mixture F-12 (DMEM/F12) culture media supplemented with 10% fetal bovine serum and 1% penicillin/streptomycin and incubated in 5% CO_2_, 95% air at 37 °C. The cell culture medium was changed daily, and all cells used were between passages 3 and 10.

### 3.2. HCG Measurement

To assess the effects of rapamycin or siRNA transfection on the endocrine activity of JEG-3 cells, the release of hCG by JEG-3 cells into the culture medium was measured when the cells were harvested, using a commercial radioimmunoassay kit (Sino-uk institute of biological technology, Beijing, China), which detects the β-subunit of hCG.

### 3.3. CCK Assay

Cell proliferation was assayed using the Cell Counting Kit-8 (CCK-8, Dojindo Labratories, Shanghai, China) according to manufacturer’s instructions. In brief, the cells were plated at a density of 2.0 × 10^4^ cells/well in 96-well plates for 48 h, 10 μL CCK-8 solution was added to culture medium, and incubated for an additional 4 h. The absorbance was measured at 450 nm using an automated microplate reader (Molecular Devices, Sunnyvale, CA, USA). The following formula was used to determine cell growth ratio values: 100 × (A450 (sample, T) − A450 (sample, T_0_))/(A450 (control, T) − A450 (control, T_0_)).

### 3.4. mTORC1 Inhibition Mediated by Rapamycin Treatment

JEG-3 cells were seeded in 6-well plates at a density of ~2.0 × 10^6^ cells/well. After 18 h in culture, the cells were incubated with 100 nM rapamycin (Gene Operation, Ann Arbor, MI, USA), a specific mTORC1 inhibitor, or the vehicle (0.02% DMSO). Then, after 48 h of rapamycin treatment, the cells were collected for mRNA extraction or western blotting analysis.

### 3.5. RNA Interference-Mediated Silencing of mTORC1

JEG-3 cells were seeded in 6-well plates at a density of ~3.0 × 10^6^ cells/well. Lipofectamine 2000 transfection reagent (Invitrogen, Carlsbad, CA, USA) and a small interfering RNAs (GenePharma, Shanghai, China) targeting raptor (100 nM; sense: 5′-CGAGAUUGGACGACCAAAUTT-3′) or a non-coding scrambled sequence (100 nM; sense: 5′-UUCUCCGAACGUGUCACGUTT-3′) was added to the culture medium at 18 h in culture. After an additional 48 h in culture, the efficiency of target silencing was determined at the protein level using western blotting.

### 3.6. mRNA Isolation and Quantitative Real-Time PCR

RNA was extracted from JEG-3 cells using TRIzol reagent (Invitrogen, Carlsbad, CA, USA). cDNA was synthesized from 1 μg of total RNA using the PrimeScript 1st Strand cDNA Synthesis Kit (Takara Bio Inc., Dalian, China) following the supplier’s instructions. Real-time PCR reactions were performed in 20 μL mixtures using the SYBR *Premix Ex Taq*™ II Kit (Takara Bio Inc., Dalian, China), containing 1 μL cDNA template, 0.5 μM forward and reverse primer, and 10 μL of SYBR *Premix Ex Taq*™ II. The gene expression was assayed in duplicate and normalized against β-actin. The relative expression values were calculated by ΔΔ*C*_t_ method of relative quantification using the Applied Biosystems 7500 Real-Time PCR System.

### 3.7. Western Blot Analysis

JEG-3 cells were washed in ice-cold phosphate buffer saline (PBS) and lysed in Radio Immunoprecipitation Assay (RIPA) buffer containing protease inhibitors and phosphatase inhibitors (50 mM Tris, 150 mM NaCl, 1% NP-40, 0.5% sodium deoxycholate, 0.1% sodium dodecyl sulfate (SDS), 1 µM aprotinin, 100 µM leupeptine, 1 mM phenylmethylsulfonylfluoride, 1 mM sodium orthovanadate, 1 mM sodium pyrophosphate, and 1 mM β-glycerophosphate). Total protein (40 μg) was separated using 10% SDS-polyacrylamide gel electrophoresis and then transferred onto a nitrocellulose membrane (PALL Corporation, New York, NY, USA). The membranes were blocked with 5% bovine serum albumin (BSA) dissolved in Tris-buffered saline containing 0.1% Tween 20. The membranes were then incubated with the appropriate primary antibody overnight at 4 °C followed by an horseradish peroxidase (HRP)-conjugated goat anti-rabbit IgG antibody (Santa Cruz Biotechnology, Santa Cruz, CA, USA) for 1 h at room temperature. The antibodies directed against phosphorylated S6K1 (Thr389) and phosphorylated 4E-BP1 (Thr37/46) were purchased from Cell Signaling Technology (Danvers, MA, USA), and an antibody against GLUT3 was obtained from Santa Cruz Biotechnology. The antibodies against total 4EBP1 and S6K1 were purchased from Sangon Biotech (Shanghai, China). The protein bands were visualized using Pierce ECL Western Blotting Substrate (Thermo Scientific, Waltham, MA, USA). The relative density of bands was assessed by densitometry using an Alpha Imager (Alpha Innotech Corporation, San Leandro, CA, USA). Target band densities were normalized against β-actin for loading controls and internal controls for blot comparison.

### 3.8. Immunofluorescence

JEG-3 cells were seeded in 24-well plates containing poly-lysine-coated coverslips and transfected with the scrambled or raptor-specific siRNA. At 24 h after transfection, the cells were fixed with methanol at −20 °C for 5 min. After permeabilization with Triton X-100, the cells were blocked in goat serum and then incubated with anti-GLUT3 antibody overnight at 4 °C. After several washes, the cells were incubated with fluorescein isothiocyanate (FITC)-conjugated goat anti-rabbit IgG for 1 h at 37 °C, and then with dye 4,6-diamido-2-phenylindole (DAPI) for 15 min to counterstain nuclei. After three washes in PBS, coverslips were mounted in a drop of 30% glycerol. Images were acquired with a NikonE800 fluorescence microscope (Nikon corporation, Tokyo, Japan).

### 3.9. Data Presentation and Statistics

The data from triplicate experiments are presented as mean ± standard deviation (SD) and were analyzed by SPSS 17.0 software (SPSS Inc., Chicago, IL, USA). To evaluate differences between groups, either the nonparametric Kruskal-Wallis test (CCK assay and hCG measurements) or Mann-Whitney *U*-test (quantitative PCR and Western blot analysis) was used. Differences with *p* < 0.05 were considered to be statistically significant.

## 4. Conclusions

In the current study, we demonstrated that changes in placental mTORC1 signaling have marked effects on the placental glucose transporter isoform-3, which may be involved in the pathophysiology of abnormal fetal growth. Because the placental mTORC1 activity and the levels of GLUT3 are shown to decrease in human IUGR associated with abnormal fetal growth, our findings provide evidence that trophoblast mTORC1 signaling inhibition may contribute to the abnormal fetal growth by decreasing GLUT3 expression. Although there are many other isoforms of trophoblast glucose transporters, our preliminary studies have indicated that the levels of expressions of the glucose transporter isoforms 1, 4 and 12 mRNAs are also down-regulated in response to rapamycin and raptor silencing (data not shown). To strengthen our understanding that maternal nutrients and fetal growth are linked by placental mTORC1 signaling, further studies including glucose transporter activity measurements are required to investigate the role of mTORC1 signaling in the regulation of transplacental glucose transport.
